# Cholera forecast for Dhaka, Bangladesh, with the 2015-2016 El Niño: Lessons learned

**DOI:** 10.1371/journal.pone.0172355

**Published:** 2017-03-02

**Authors:** Pamela P. Martinez, Robert C. Reiner, Benjamin A. Cash, Xavier Rodó, Mohammad Shahjahan Mondal, Manojit Roy, Mohammad Yunus, A. S. G. Faruque, Sayeeda Huq, Aaron A. King, Mercedes Pascual

**Affiliations:** 1 Department of Ecology and Evolution, University of Chicago, Chicago, Illinois, United States of America; 2 Department of Epidemiology and Biostatistics, Indiana University Bloomington School of Public Health, Bloomington, Indiana, United States of America; 3 Center for Ocean-Land-Atmosphere Studies, George Mason University, Fairfax, Virginia, United States of America; 4 Catalan Institution for Research and Advanced Studies (ICREA), Catalunya, Spain; 5 Climate and Health Program, ISGlobal, Catalunya, Spain; 6 Institute of Water and Flood Management, Bangladesh University of Engineering and Technology, Dhaka, Bangladesh; 7 Department of Ecology and Evolutionary Biology, University of Michigan, Ann Arbor, Michigan, United States of America; 8 International Centre for Diarrheal Disease Research, Dhaka, Bangladesh; 9 Department of Mathematics, University of Michigan, Ann Arbor, Michigan, United States of America; 10 Santa Fe Institute, Santa Fe, New Mexico, United States of America; Columbia University, UNITED STATES

## Abstract

A substantial body of work supports a teleconnection between the El Niño-Southern Oscillation (ENSO) and cholera incidence in Bangladesh. In particular, high positive anomalies during the winter (Dec-Feb) in sea surface temperatures (SST) in the tropical Pacific have been shown to exacerbate the seasonal outbreak of cholera following the monsoons from August to November. Climate studies have indicated a role of regional precipitation over Bangladesh in mediating this long-distance effect. Motivated by this previous evidence, we took advantage of the strong 2015–2016 El Niño event to evaluate the predictability of cholera dynamics for the city in recent times based on two transmission models that incorporate SST anomalies and are fitted to the earlier surveillance records starting in 1995. We implemented a mechanistic temporal model that incorporates both epidemiological processes and the effect of ENSO, as well as a previously published statistical model that resolves space at the level of districts (thanas). Prediction accuracy was evaluated with “out-of-fit” data from the same surveillance efforts (post 2008 and 2010 for the two models respectively), by comparing the total number of cholera cases observed for the season to those predicted by model simulations eight to twelve months ahead, starting in January each year. Although forecasts were accurate for the low cholera risk observed for the years preceding the 2015–2016 El Niño, the models also predicted a high probability of observing a large outbreak in fall 2016. Observed cholera cases up to Oct 2016 did not show evidence of an anomalous season. We discuss these predictions in the context of regional and local climate conditions, which show that despite positive regional rainfall anomalies, rainfall and inundation in Dhaka remained low. Possible explanations for these patterns are given together with future implications for cholera dynamics and directions to improve their prediction for the city.

## Introduction

Climate variability, specifically the El Niño-Southern Oscillation (ENSO), has been shown to influence the year-to-year variation of seasonal outbreaks of cholera in Bangladesh [1–6]. In particular, interannual variations of the disease have been shown to be positively associated with sea surface temperatures (SST) in the Niño3.4 region of the tropical Pacific (5N-5S, 170W-120W), an area that warms substantially during El Niño events [2]. Regional rainfall was identified as one of the local drivers that mediates this long-range effect of ENSO [7], with evidence for an influence of high monsoon rains and river discharge on disease levels in Bangladesh (e.g. [8–14]).

Both statistical analyses and process-based mathematical models for the population dynamics of the disease have been applied to understanding the influence of ENSO on rural areas of Bangladesh (e.g. [2, 5]). For the city of Dhaka itself, the capital of Bangladesh, application of mechanistic models incorporating epidemiological processes is still lacking, although a more phenomenological approach has been developed to analyze the spatio-temporal transmission dynamics at the level of “thanas” or districts within the city [15]. The statistical model of Reiner *et al*. (2012) [15] specifically identified the existence of two distinct spatial regions within Dhaka based on the dynamics of the disease: the truly urban core of the city and its more rural periphery, with the former exhibiting much higher disease incidence and a stronger response to ENSO. This distinction between the core and periphery of the city was also found to be relevant for rotavirus, suggesting that the causal links between climate and disease are a more general feature of diarrheal diseases in this region, independent of their particular transmission pathways [16]. A similar conclusion is supported by comparisons between cholera and shigellosis regarding their association with flooding and SST in the Pacific [17].

**Fig 1:**
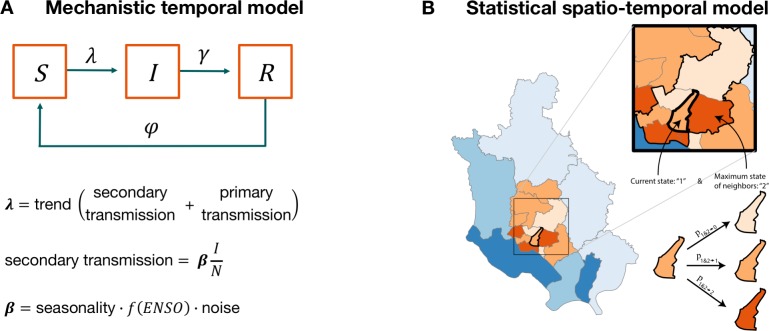
Schematic representation of the models. (A) Mechanistic temporal model. The population is divided into three classes, for the susceptible *S*, infected *I* and recovered *R* individuals. The arrows denote rates of flow among these classes. The force of infection λ includes three components: a long-term trend, secondary transmission that depends on the levels of infection in the population, and primary transmission at a constant rate from an environmental reservoir of the pathogen. The transmission coefficient (or rate) β in secondary transmission incorporates seasonality, interannual variation as a function of the ENSO index, and environmental noise. (B) Statistical spatio-temporal model. Districts of the city known as “thanas” are grouped into two main regions (depicted in orange for the core and in blue for the periphery). Thanas within the same group follow the same dynamical rules in terms of transitions between cholera levels or states from one month to the next. Three states are considered and used to discretize the case data: no cholera (0), low cholera (1), or high cholera (2) as indicated by the different color intensity. The probability of transition between states from one month to the next depends on the season, the maximum state of neighboring districts, and the climate covariate (ENSO). For details on the models, see Methods and S1 Text.

Recently, new statistical approaches for inferring model parameters based on time series data have allowed for flexible representations of mechanisms that more closely represent epidemiology in models that also incorporate both measurement and process noise [18,19]. Although these process-based models have been applied to glean insight into epidemiological processes in a number of infectious diseases from retrospective data, they have been used only in a few exceptions to generate and evaluate forecasts (e.g. [20, 21]). Motivated by the large El Niño of 2015–2016, we examine here the ability of a process-based (mechanistic) model to predict cholera incidence in Dhaka by combining epidemiology and climate variability (Fig 1A). We also compare the results to those obtained with the previously published statistical model for cholera in the city (Fig 1B). Based on our previous modeling work, we chose the Niño3.4 index to characterize the El Niño event. Previous work has shown that correlations between Bangladesh cholera and winter SST are statistically significant across the entire Niño3.4 domain [7,17]. The Niño3.4 value observed in the region for January 2016 (2.60) is comparable in magnitude to the one observed for the large El Niño event of 1998 (2.56), when Dhaka suffered the largest cholera outbreak in the last 20 years and one of the worst floods in its history affecting more than 50% of the city's area [22]. We focus on cholera reported cases from the core of the city for the process-based model, based on the heightened sensitivity to ENSO in this region, the low number of cases in the periphery and its low contribution to the force of infection in the core [15, 23]. The monthly surveillance data are divided into a training set from January 1995 to December 2010, used to fit the mechanistic model, and an “out-of-fit” set from January 2011 to October 2016, used to evaluate the predictability of the model. For the statistical model, the out-of-fit set starts in 2009, based on the published fit [15]. We specifically ask whether these two models can be used as an early-warning system, able to forecast cholera outbreaks following the monsoon season, based on observed January Niño3.4 values and on previous cholera cases. We then discuss our results, both true negative predictions up to 2015 and a false positive prediction for 2016, in the context of precipitation and inundation records at regional and local levels. We consider open questions raised by these findings on the changing connections between global, regional and local climatic conditions, and on implications for the future of cholera risk and its prediction for the city.

## Results

**Fig 2:**
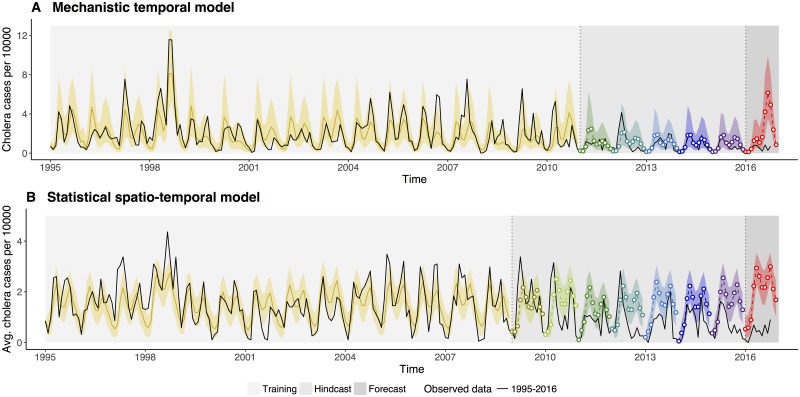
Comparison of simulated and predicted monthly cases with those reported for Dhaka, Bangladesh. The observed cases per 10000 individuals are shown in black for the core of the city (A) and for the average of all thanas (for core and periphery, (B)). The median of 1000 simulations is shown in dark yellow, with the 10–90% confidence intervals (C.I.) in the shaded lighter color. Simulations of the predicted out-of-fit data are shown in different colors starting in 2011 in (A) and in 2009 in (B), with the median for each month in open circles, and their respective C.I. envelope in a shaded lighter color. Vertical dotted lines and corresponding background shading indicate three different kinds of model simulations and “predictions”: for the initial period (white background), the model is simulated from 1995 forward. Thus, the comparison to data is not based on the typical next-step (next-month) prediction for which it is somewhat trivial for most models to capture the fitted data. These simulations span more than a decade. The second period (light gray background) shows predictions for out-of-fit years, with simulations starting in January and spanning the whole year. We refer to these predictions that cover windows of time in the past, as hindcasts. Finally, the last period is for the current year and constitutes a true forecast (for the upcoming fall season) which we implemented in ‘real time’ and compared to observations once the cholera season had passed.

Normalized incidence data exhibit strong interannual variability (Fig 2). The seasonality in cholera incidence is bimodal with two peaks per year: in spring and in autumn, preceding and following the monsoons respectively. For the mechanistic model, the observed and the simulated data exhibit yearly peaks and troughs that fall in the same months for most years, capturing the bi-modal seasonal patterns reasonably well (Fig 2A). Such agreement is encouraging given that these simulations are not one time-step ahead predictions but instead, 15-yr long trajectories starting from estimated initial conditions.

The predicted yearly data for the period 2011–2016 were generated by updating the initial states of the epidemiological variables for January each year, as well as the estimated values of the parameters for each year starting in 2011. Model predictability was evaluated for the years between 2011 and 2016. Because the model is stochastic (incorporating noise in the dynamics), we generated both the median value and the 10–90% confidence intervals of 1000 predictions for each month. For the most part the median prediction appears close to the data, with the observed cases falling within the uncertainty of the confidence intervals. This result suggests that cholera incidence is forecasted accurately, including the decline observed in the data until 2015 and the low levels of incidence characteristic of the past 5 years. We note a slight over-prediction of some of the fall seasons (2012 and 2015) which exhibited almost no cases those years in a somewhat uncharacteristic seasonal pattern. Similarly, simulations of the statistical model show that the predicted cases closely match the observed data within the training set as already shown in Reiner *et al*. (2012) [15] for data up to 2009 (Fig 2B). Here, we can evaluate the ability of this model to predict the new data not used to fit the model, which has become available since that study. For the most part, the predictions produce the observed lower levels of cholera, with an overprediction of the same fall peaks for 2012 and 2015. This overprediction is larger possibly because the spatio-temporal model does not incorporate a long-term trend.

To more formally evaluate predictability, we considered the probability of exceeding a threshold number of cases for the whole post-monsoon cholera season (Aug–Dec), with the threshold based on the distribution of outbreak size in the data. We are interested here in evaluating the ability of the model to predict the occurrence vs. the lack of “large” outbreaks, given their relevance to public health and in light of the high El Niño index reported for January 2016. In Table 1, the probability of cholera incidence exceeding the 50th, 75th and 95th percentiles for 2011–2015 are reported, with these different thresholds representing outbreaks of increasingly higher magnitude. Results from the mechanistic model indicate the absence of a large outbreak, consistent with the observed data for that period, with none of the years exhibiting a probability greater than 50% of exceeding the thresholds (Table 1). In other words, the model would have accurately predicted the low risk of cholera in the past five years. Likewise, the statistical model shows consistent results for the most part, with a probability higher than 50% of being above the median for only one of the years (2015). Otherwise for the 75% and 95% thresholds, the probabilities of exceeding them are consistently low. For the fall season of 2016, the same forecast analysis reveals probabilities of 87% and 98% for the mechanistic and statistical models respectively, of a large outbreak (defined as surpassing the 75th quantile of outbreak size). Similar results for 2016 are observed when considering a mechanistic model that includes cases from both regions of the city (core and periphery, S1 Fig). These results imply a false positive prediction when compared to the observation of a low cholera season based on surveillance data for Aug-Oct 2016.

**Table 1:** Hindcasts for the indicated years and forecast for 2016, for the post-monsoon (Aug-Dec) season of cholera. The distribution of observed cases for this same post-monsoon period for the training data used to fit the models was used to estimate the values of the 50th (the median), 75th and 95th quantiles. These values are used as thresholds to define outbreaks of increasing size: a season that exceeds the median is considered anomalous, one that exceeds the 75% level, is considered a large outbreak, and one that exceeds the 95% level, an extreme outbreak. The average observed cases for each year are shown next with an indication of whether they exceed the threshold (yes, “outbreak”) or not (no, “no outbreak”). The proportion of 1000 simulations that fall above each threshold level is reported as a probability, and a probability > 50% is interpreted as a prediction of an outbreak, specified in the last column.

Year	Quantile	Mechanistic temporal model			Statistical spatio-temporal model		
		Observed	Probability	Prediction (prob. > 50%)	Observed	Probability	Prediction (prob. > 50%)
2011	50	no	14.3	no	no	2.7	no
2011	75	no	1.3	no	no	0.5	no
2011	95	no	0.1	no	no	0.0	no
2012	50	no	17.6	no	no	17.7	no
2012	75	no	2.0	no	no	5.1	no
2012	95	no	0.1	no	no	0.0	no
2013	50	no	19.0	no	no	43.1	no
2013	75	no	1.5	no	no	21.2	no
2013	95	no	0.0	no	no	0.1	no
2014	50	no	18.7	no	no	33.8	no
2014	75	no	1.6	no	no	14.2	no
2014	95	no	0.0	no	no	0.0	no
2015	50	no	18.3	no	no	56.6	yes
2015	75	no	1.8	no	no	32.0	no
2015	95	no	0.1	no	no	0.1	no
**2016**	**50**	**--**	**99.0**	**yes**	**--**	**99.6**	**yes**
**2016**	**75**	**--**	**86.5**	**yes**	**--**	**97.6**	**yes**
**2016**	**95**	**--**	**50.1**	**yes**	**--**	**27.2**	**no**

**Fig 3:**
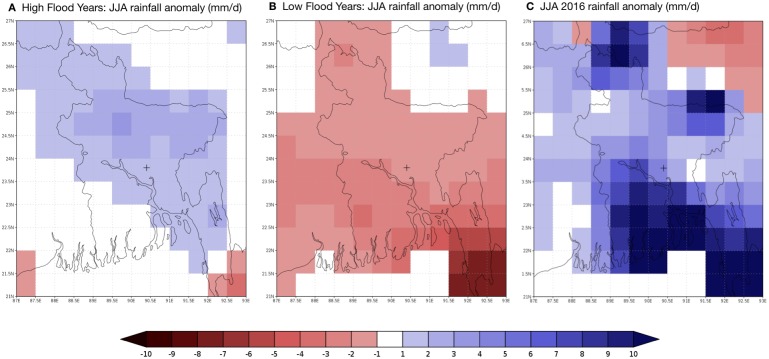
Deviation from climatological June-August rainfall for (A) five high flooding years, (B) five low flooding years, and (C) 2016. Units are (mm/d). The cross symbol indicates the location of Dhaka.

This discrepancy motivates consideration of precipitation and inundation patterns at both regional and local scales. The evolution of the monsoon over Bangladesh is examined based on the Climate Prediction Center Unified (CPC_UNI) global daily precipitation data set [24, 25], which covers the period 1979-August 2016 and is updated daily in real time. We compare the evolution of the 2016 monsoon with previous high and low flooding years, all following positive DJF mean Niño3.4 anomalies as determined by the National Weather Service (NWS) Climate Prediction Center (CPC). Five high flooding (1987, 1988, 1998, 2004, 2007) and five low flooding (1983, 1992, 1995, 2003, 2005) years were selected, and the average difference in June-August rainfall relative to the 1979–2015 mean is shown in Fig 3. We find that for the high flooding years, June-August rainfall was higher than normal across much of Bangladesh (Fig 3A), while in the low flooding years, June-August rainfall was below normal across the entire region (Fig 3B). Data from the CPC also indicates rainfall across Bangladesh and its immediate surroundings was above normal for the 2016 June-August period, consistent with a ‘high flood’ response (Fig 3C). When compared to 2007 when Bangladesh had significant and destructive flooding, however, the 2016 rains are significantly less (not shown). Although the 2016 anomalies are more comparable to those for 1998, there was less rain to the north and more to the south of Dhaka in 2016 relative to 1998. We also note that signals early in the monsoon season (June) were also more mixed for 2016 (S2 Fig). Despite the five high and low flood years showing June anomalies consistent with those of the full monsoon season, the current year exhibited above normal rainfall across the Northwest India and northern Bangladesh and below normal rainfall across the center of the country.

At the local level, we found that the total precipitation in Dhaka for Jun-July 2016 was below average compared to the past 9 years (S3 Fig), consistent with the low percentage of flooding observed in this region during August 2016 (less than 1% of the city, Humanitarian Response Plan: Bangladesh Monsoon Floods August 2016). In addition, tide levels at two stations used to determine flood conditions within the city also indicate a lack of inundation (S4 Fig).

## Discussion

The El Niño-Southern Oscillation (ENSO) acts as the main driver of interannual climate variability worldwide. As such, the associated anomalies in sea surface temperatures in the tropical Pacific provide a basis for understanding the interannual variability of several phenomena around the globe, including that of climate-sensitive infectious diseases, such as water-borne and vector-borne infections. Here, we implemented a mechanistic transmission model that incorporates both epidemiological processes and the effect of ENSO, as well as a previously published statistical model, to forecast cholera risk in the city of Dhaka for the upcoming fall season based on observed SST anomalies in the tropical Pacific. The models produced accurate hindcasts for the low-cholera years preceding 2016 but failed to predict the lack of an outbreak in response to the strong 2015–2016 El Niño.

The predictive ability of the models was evaluated based on the incidence of the last five years. Given the uniformly low levels of cholera during that period, this assessment concerns only true or false negatives, that is the ability of the model to predict the lack, and not the occurrence, of an outbreak. Although we correctly predict the lack of an outbreak and the overall low risk of cholera for that period, we overpredict the actual number of monthly cases during the fall season for two of the years (2012 and 2015) whose fall peaks were almost completely absent. This pattern may result from the stochasticity inherent to low levels of the disease, and in part be accounted for the long-term trend in combination with the lack of warm anomalies in ENSO conditions. The statistical model overpredicts these recent low seasons further, possibly because it does not incorporate such a trend. We note that in general, this model also tends to underpredict the large events (like 1998) because of the transformation from actual cases to three discrete levels which reduces the tail of the distribution of cases (a bias that can be corrected for systematically as explained in [15]).

For 2016, both models predicted a high risk of a large outbreak based on the magnitude of the current El Niño condition, similar to that observed for 1998 (with or without the bias correction for the statistical formulation). Predictions that involve long-distance connections, such as ours, trade the advantage of a long lead time (from 9 to 11 months here) with the inherent limitation that an El Niño event is not fully characterized by the magnitude of the tropical Pacific SST anomalies alone, and that ultimately, the regional changes in the climate variability of Bangladesh, and the more local changes in the rainfall and inundation patterns of Dhaka, are what matters to the population dynamics of the disease. Given that some strong El Niño events have resulted in low rainfall and low flooding in Bangladesh in the past, and the somewhat anomalous impact of the 2015–2016 event on rainfall in other regions (such as California), it is important to recall that no ENSO index alone can fully capture the impact of a given event. Significant difference in impact over Bangladesh from events characterized by similar index values have occurred in the past, and regional rainfall for the country is influenced by SST conditions in other parts of the global ocean, in the Pacific and the Indian Oceans [26, 27].

We rely here on observations on the regional and local climatic conditions to consider possible explanations for the lack of an outbreak. Although preliminary, these observations on the temporal variability of rainfall and inundation suggest non-stationary hydrological conditions relevant to the connection between ENSO and cholera in Dhaka. The lack of flooding in the city itself (S4 and S5 Figs), despite reports of inundation in other parts of the country, provide a proximate causal break in the connection between SSTs in the tropical Pacific and high cholera incidence in Dhaka. Although low tide levels are consistent with low rains in Dhaka itself, it is interesting to note that recent years with high rains (such as 2007 and 2015) did not result in flooding either (S5 Fig). Importantly, this discrepancy with the tighter connection between rains and floods in the 1990s (e.g. 1998), suggests improved flood control in the city [28]. Further evidence for such a change in the local hydrology is provided by the observation that regional rainfall anomalies were positive (Fig 3), albeit with a late start of the season and a geographical distribution to the North and South of the city that may also contribute to low water levels in Dhaka. Given that the Brahmaputra is a transboundary river and Bangladesh is a lower riparian country of the basin, we would expect water level at the gauge station during the monsoon to be controlled by the upstream flow of the Brahmaputra basin and the regional rainfall within the country.

Flood control however may not be the whole story. The fresh water inflow to Dhaka from upstream is decreasing due to gradual siltation, encroachment, connectivity loss, and other modifications of the river. Another striking pattern is the decreasing trend in both rainfall and tide levels for the recent decade. Thus, Dhaka appears in the midst of a dry decadal spell relative to average conditions, coincident with low cholera levels and the lack of a response to the 2015–2016 El Niño. Under this trend, even in the presence of anomalous high rains, flood conditions and the associated disease response to El Niño may not develop.

A further, more intriguing, contribution to the low response could be that the prolonged hiatus in large El Niños of the last decade associated with the sustained window of many successive years with low incidence, have diminished the environmental reservoir of the bacterium *Vibrio cholerae* in its pathogenic form. Under this scenario, the system may be poised at biological conditions that render a response to climate events in the coming future more unlikely.

A major question that follows is whether Dhaka is therefore set in a course of diminishing cholera risk regardless of global and regional climatic conditions, or whether the city might experience instead a disease resurgence if climate events that facilitate cholera (El Niño and flooding) did enter a more active period. Answers to this question will require a better understanding of the preliminary patterns in local and regional hydrology discussed here, patterns of relevance to public health beyond cholera.

More specifically, this work raises the question of ‘what next’ on forecasting cholera with transmission models. As mentioned before, reliance on a teleconnection (the effect of ENSO) allows a forecast with a considerable lead time, and as such, provides an early warning for the public health system to pay close attention to the evolution of the monsoon and associated floods in a given year. Going forward, the event of 2015–2016 will contribute to improving the models by providing information on a second large El Niño event and a different outcome than that of 1998. The consideration in the models of additional aspects of the global climate that can potentially modulate the relationship between ENSO and regional precipitation in Bangladesh is also worth exploring, including the persistence of warm SST anomalies in the central Pacific, and SST anomalies in the Indian Ocean (the Indian Ocean Dipole, IOD) which exhibit a non-stationary association with cholera in Dhaka [27, 29]. More importantly, it is clear that the forecasting system should operate in tandem with a second-stage family of models that relies on local climate conditions and provides a much shorter lead time (of at most a month). Improvements in the understanding of the variability and non-stationary connections between ENSO and the monsoons for this region, will also further inform cholera models. This ‘real-time’ forecasting exercise has underscored the challenges of prediction in epidemiological-climate systems which are inherently non-stationary [29, 30], but has also raised the optimistic possibility that cholera risk in Dhaka is declining, including a decreased sensitivity to ENSO.

## Methods

The original cholera data consist of daily cases from Jan 1995 to Oct 2016 obtained from the ongoing surveillance program by the International Center for Diarrheal Disease Research, Bangladesh (icddr,b), in which a systematic subsample of all patients visiting the hospital, which serves as the main treatment center for the greater Dhaka city area, is tested for cholera. Reported cases were added by month, per “thana” or administrative subdivision. For each thana, the population was computed by interpolating the three decadal censuses beginning in year 1991, 2001 and 2011. Cases were then aggregated over the thanas comprising two regions, core and the periphery of the city, according to the partition proposed by Reiner *et al*. (2012) [15].

The process-based model follows the temporal changes in the number of cases in the core of the city. It consists of a Susceptible-Infected-Recovered-Susceptible (SIRS) compartmental formulation in which the population is subdivided into the following classes: *S* for susceptible individuals, *I* for infected and infectious individuals, and *R* for recovered individuals who have acquired immunity to the disease. Acquired immunity is temporary and wanes at rate ϕ as individuals return to the *S* class. The recovery rate γ of infected individuals and other parameters are estimated using a recently developed likelihood-based inference method (see S1 Text for model details). The rate of transmission per susceptible individual (or force of infection λ) contains three components: a long-term trend, a representation of secondary transmission (depending on the level of infected individuals in the population), and primary transmission (at a constant rate from environmental reservoir). Primary transmission is intended to represent here new cases arising from contact with a reservoir of the pathogen in the environment that is effectively decoupled from levels of infection in the population (i.e. has no memory of these levels) and provides a background source of infection. By contrast, secondary transmission depends on the proportion of the population that is infected (I/N), although we note that the transmission process may still involve water bodies in the environment, as for simplicity the model does not explicitly differentiate this route from more direct transmission within homes (e.g. through food). The expression for secondary transmission includes the coefficient or transmission rate β including a seasonal component, the interannual effect of ENSO, and environmental noise (Fig 1 and S6 Fig). The effect of ENSO is included as a function of SST anomalies for the month of January, following our earlier work showing the association of the September-December cholera season with this particular month (and with the closely related December-January-February running mean for SST anomalies in the Niño3.4 region, the Oceanic Niño Index, ONI) [7]. The model further incorporates measurement error with a reporting rate that is also estimated and accounts for under-reporting. For a full description of the model, see S1 Text.

The statistical model follows the spatio-temporal transmission dynamics of the disease at the spatial resolution of thanas or districts in the city. It categorizes the monthly thana-level case data into three states: “no cholera”; “low cholera” (cases per 10000 between 0 and 1.85); and “high cholera” (cases per 10000 above 1.85) as described in [15]. The model estimates the month-to-month transitions of each thana as a set of three transition probabilities (conditioned on the value of the current state only). These probabilities are functions of the current state of the thana, the maximum state of the neighboring thanas, the value of ENSO (climate covariate) and the season (Fig 1). We note that ENSO enters in this model as a continuous covariate for all months with a lag of 11 months. The parameter values used for these predictions were fitted to data from 1995–2008 in [15]. For complete details on the model formulation and fitting, including the categorization criterion, see [15].

## Supporting information

S1 Fig Comparison of simulated and predicted monthly cases with those reported for the whole city of Dhaka, Bangladesh. The normalized cases from the core and periphery of the city are shown in black. The median of 1000 simulations is shown in dark yellow, with the 10–90% confidence intervals (C.I.) in the shaded lighter color. The background shading rectangle corresponds to the forecast for 2016. Probabilities of surpassing the 50th, 75th and 95th quantiles are 99%, 89% and 49% respectively.
(PDF)

S2 Fig Deviation from climatological June rainfall for (A) five high flooding years, (B) five low flooding years, and (C) 2016. Units are (mm/d). (PDF)

S3 Fig Precipitation in Dhaka for the months June and July. The dotted gray line corresponds to the average precipitation during Jun–Jul for the period 2007–2015.
(PDF)

S4 Fig Water level in Dhaka. (A) Tide level from Demra, a gage station located at 23.7232 N, 90.5018 E (in the eastern part of Dhaka). (B) Tide level from Mirpur, a second gage station located at 23.7833 N, 90.3385 E (in the western part of Dhaka). The dotted lines correspond to the flooding threshold for each station (5.75m for Demra and 5.94m for Mirpur) used by the city to define flood conditions. The blue and the red lines refer to the low and high tide respectively.
(PDF)

S5 Fig Comparison between the evolution of (A) seasonality and (B) long-term variability (periods > 2 years) for rainfall (blue) and high tide level (black) at Mirpur station in Dhaka. Rainfall amounts and high tide level measures were both accumulated and a linear trend was removed by least squares estimation. Individual oscillatory components were orthogonally separated through an eigen decomposition that partitions the process in the frequency domain using a lagged covariance matrix constructed from the data (R. Vautard and M. Ghil, 1989 doi:10.1016/0167-2789(89)90077-8; X. Rodó et al 2002 doi:10.1073/pnas.182203999). Two sequential decompositions were needed in order to achieve a proper extraction and reconstruction of constituent signals, given the well-known noisy nature of rainfall time series. An augmented order of 275 was used in the decomposition to obtain a proper separation of the signals from the noise floor (given that the reconstruction of signals was performed on daily data). No significant changes were observed on a range of ±10% of the aforementioned embedding dimension. Amounts are expressed as mm.
(PDF)

S6 Fig (A) The six periodic splines considered in the seasonality component of the transmission rate. The fourth and fifth splines were used to incorporate the effect of ENSO (thicker lines). (B) Functional form of ENSO, estimated with the parameters from the MLE (red) and its confidence interval (gray shaded area). (C) Transmission rate by month. The expression for 2016 is shown in magenta, while the black line illustrates a year with ENSO ~ 0.
(PDF)

S1 Table Epidemiological parameter estimates and confidence intervals. (DOCX)

S1 Text Model equations, parameter estimation, and prediction evaluation. (DOCX)
