# Supplementary figures and images for: Cholera forecast for Dhaka, Bangladesh, with the 2015-2016 El Niño: Lessons learned
Source: PLoS One. 2017 Mar 2;12(3):e0172355. doi: 10.1371/journal.pone.0172355 (PMC5333828; doi:10.1371/journal.pone.0172355)

**Mechanistic temporal model: Core and Periphery**

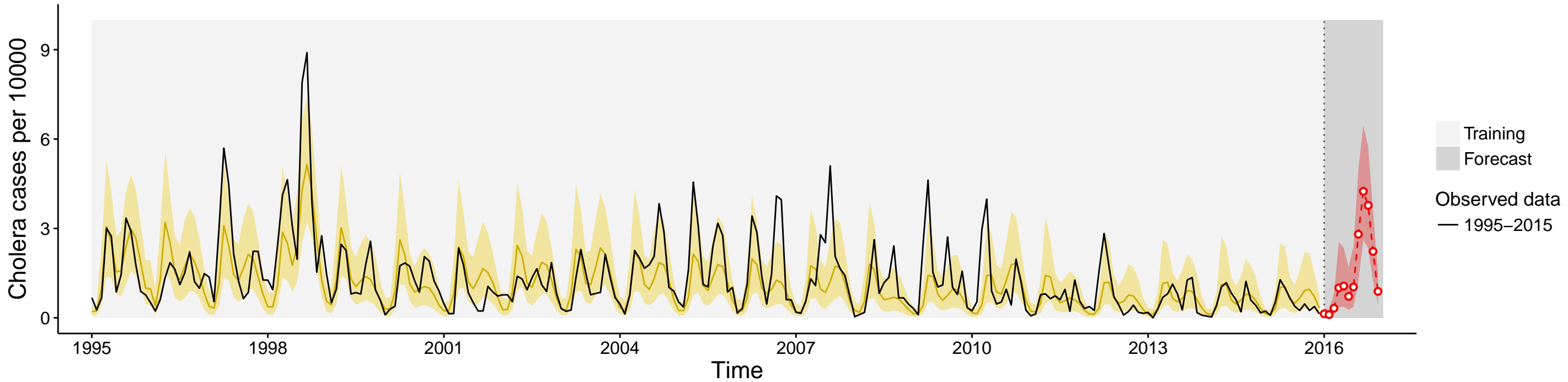

Supplement: S1 Fig — The normalized cases from the core and periphery of the city are shown in black. The median of 1000 simulations is shown in dark yellow, with the 10–90% confidence intervals (C.I.) in the shaded lighter color. The background shading rectangle corresponds to the forecast for 2016. Probabilities of surpassing the 50th, 75th and 95th quantiles are 99%, 89% and 49% respectively. (PDF) [file pone.0172355.s001.pdf]

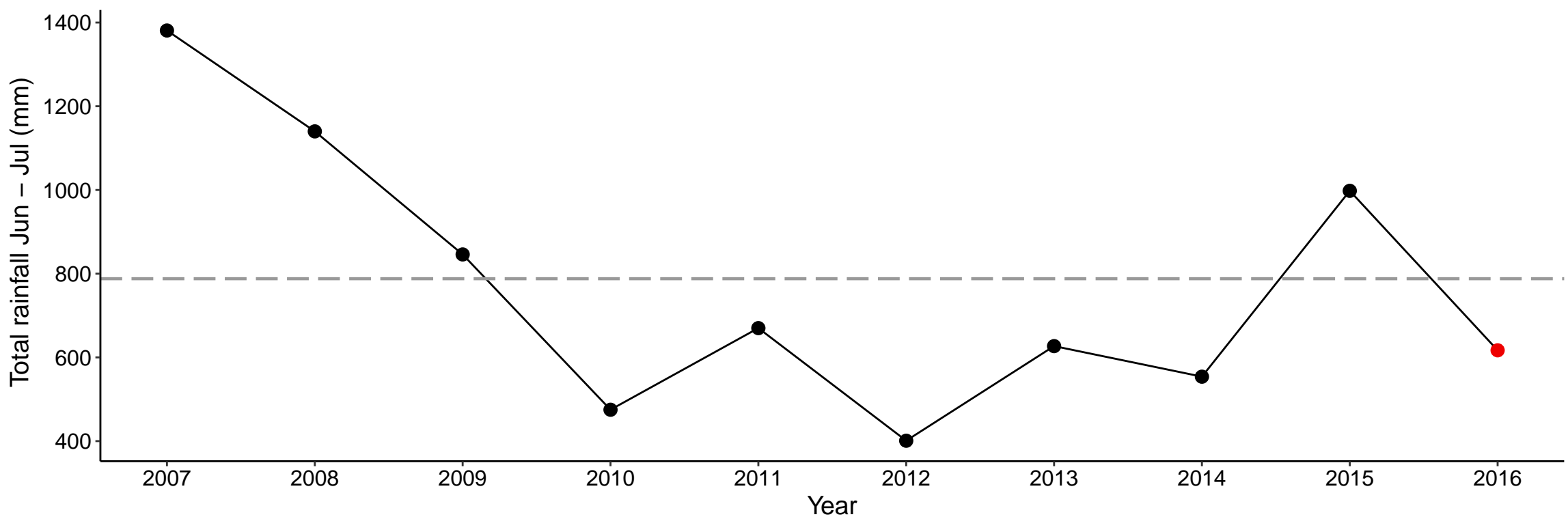

Supplement: S3 Fig — The dotted gray line corresponds to the average precipitation during Jun–Jul for the period 2007–2015. (PDF) [file pone.0172355.s003.pdf]

**A Demra station**

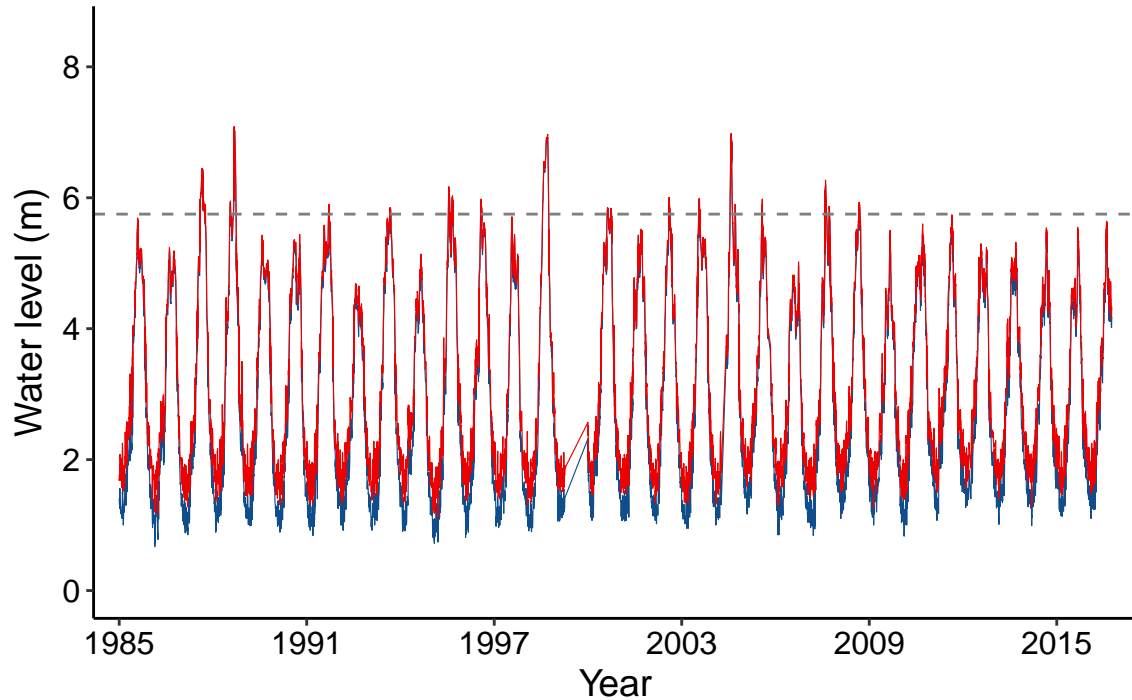

**B Mirpur station**

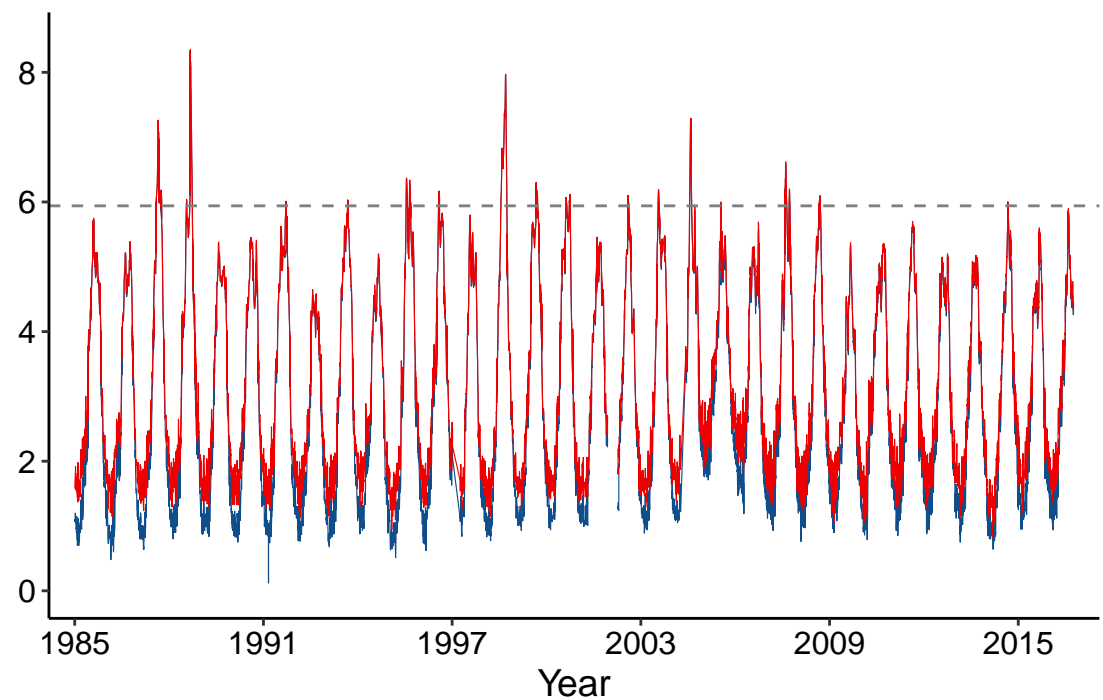

Supplement: S4 Fig — (A) Tide level from Demra, a gage station located at 23.7232 N, 90.5018 E (in the eastern part of Dhaka). (B) Tide level from Mirpur, a second gage station located at 23.7833 N, 90.3385 E (in the western part of Dhaka). The dotted lines correspond to the flooding threshold for each station (5.75m for Demra and 5.94m for Mirpur) used by the city to define flood conditions. The blue and the red lines refer to the low and high tide respectively. (PDF) [file pone.0172355.s004.pdf]

## A Seasonality

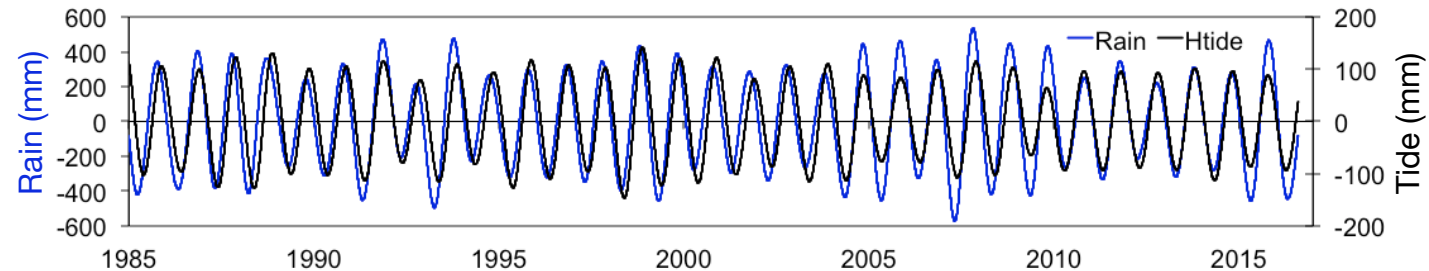

## B Long-term

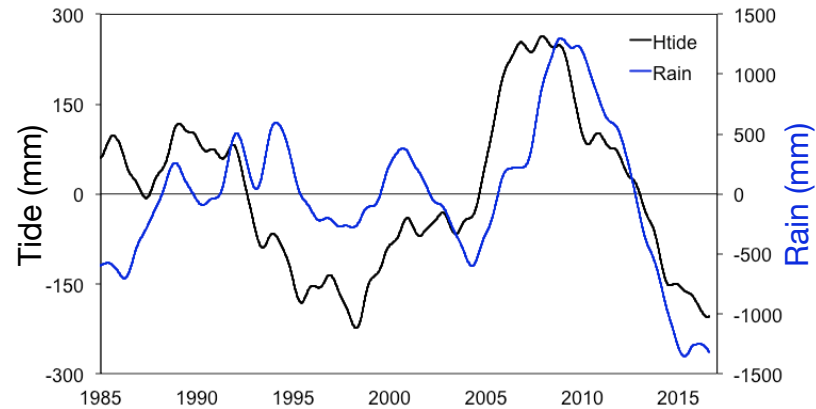

Supplement: S5 Fig — Rainfall amounts and high tide level measures were both accumulated and a linear trend was removed by least squares estimation. Individual oscillatory components were orthogonally separated through an eigen decomposition that partitions the process in the frequency domain using a lagged covariance matrix constructed from the data (R. Vautard and M. Ghil, 1989 doi:10.1016/0167-2789(89)90077-8; X. Rodó et al 2002 doi:10.1073/pnas.182203999). Two sequential decompositions were needed in order to achieve a proper extraction and reconstruction of constituent signals, given the well-known noisy nature of rainfall time series. An augmented order of 275 was used in the decomposition to obtain a proper separation of the signals from the noise floor (given that the reconstruction of signals was performed on daily data). No significant changes were observed on a range of ±10% of the aforementioned embedding dimension. Amounts are expressed as mm. (PDF) [file pone.0172355.s005.pdf]

**A**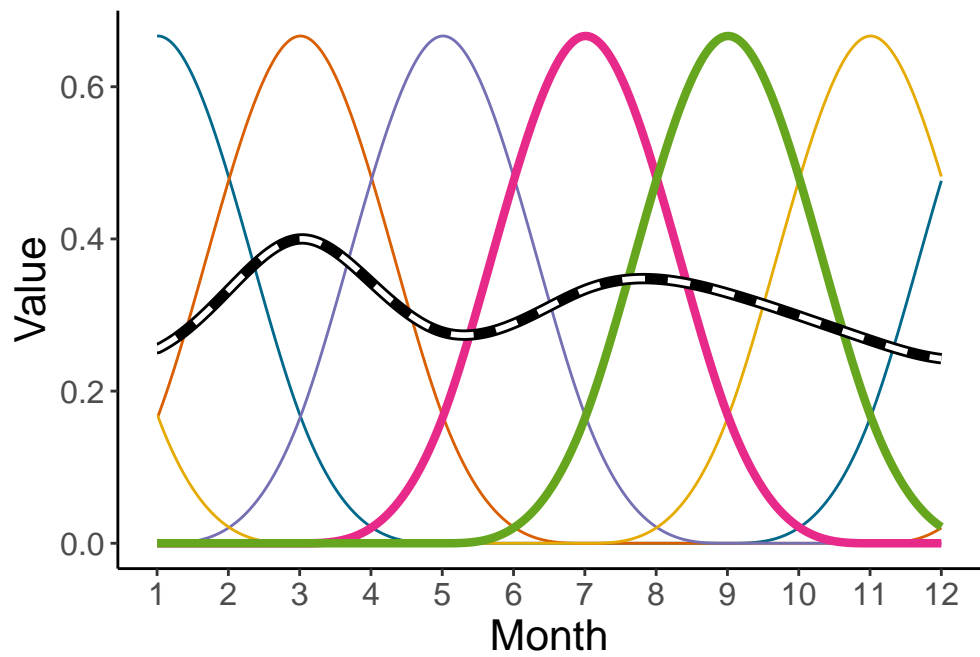**B**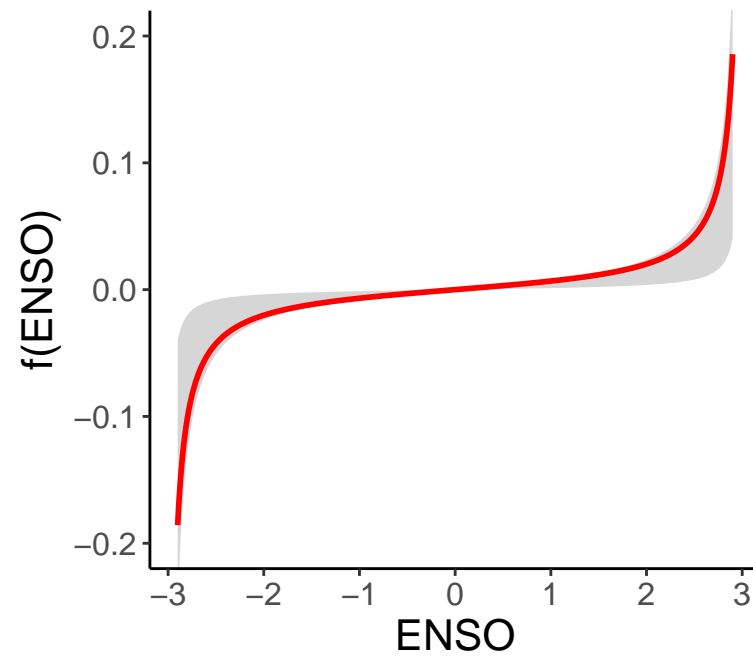**C**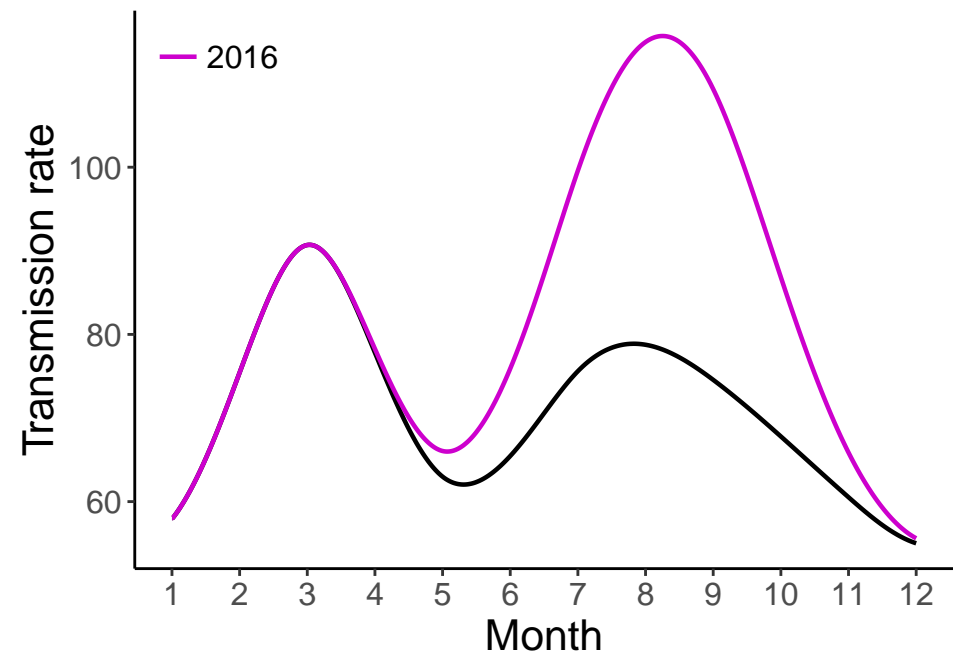

Supplement: S6 Fig — (A) The six periodic splines considered in the seasonality component of the transmission rate. The fourth and fifth splines were used to incorporate the effect of ENSO (thicker lines). (B) Functional form of ENSO, estimated with the parameters from the MLE (red) and its confidence interval (gray shaded area). (C) Transmission rate by month. The expression for 2016 is shown in magenta, while the black line illustrates a year with ENSO ~ 0. (PDF) [file pone.0172355.s006.pdf]
