# Supplementary material for: Cholera forecast for Dhaka, Bangladesh, with the 2015-2016 El Niño: Lessons learned
Source: PLoS One. 2017 Mar 2;12(3):e0172355. doi: 10.1371/journal.pone.0172355 (PMC5333828; doi:10.1371/journal.pone.0172355)
Supplement: S2 Fig — (PDF) [file pone.0172355.s002.pdf]

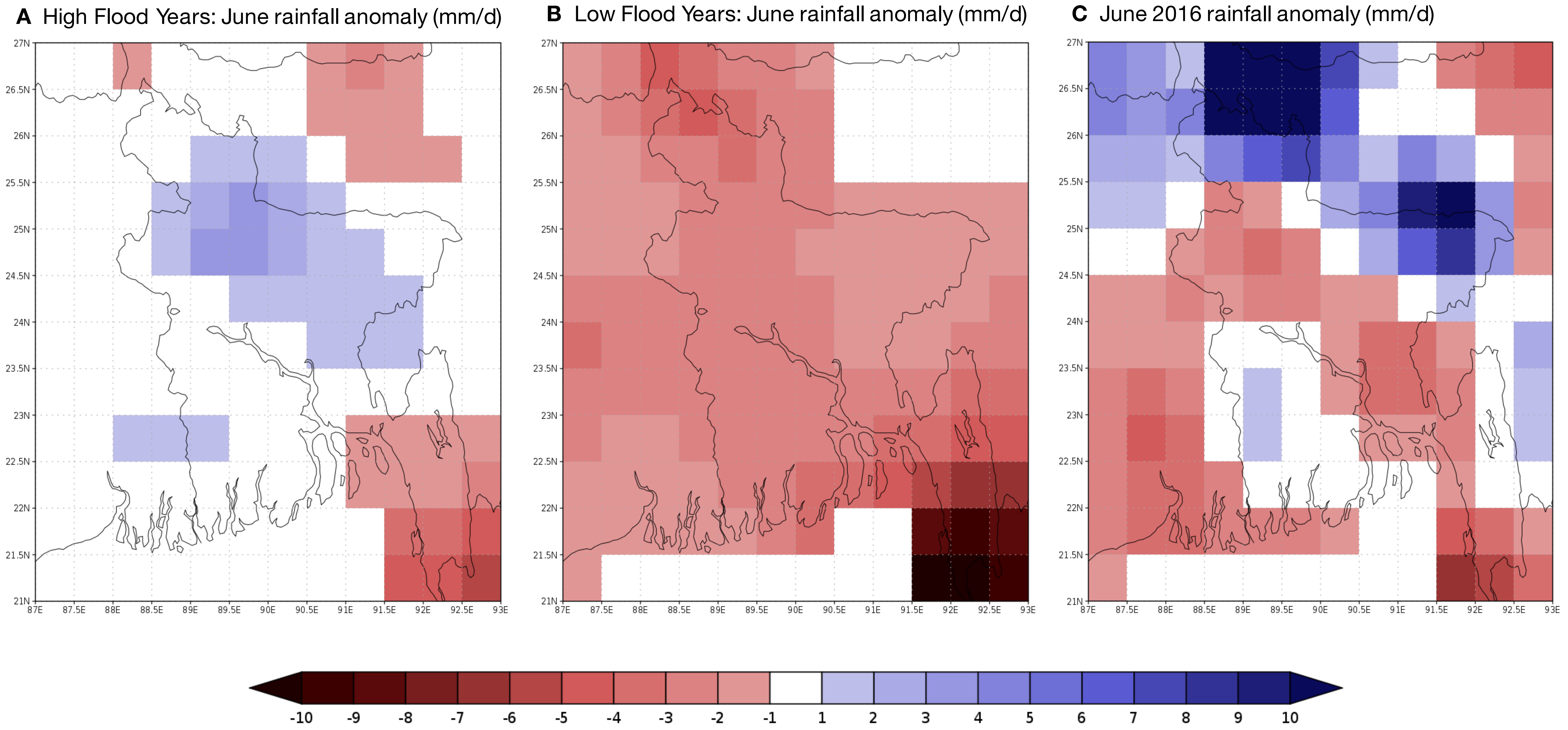

A High Flood Years: June rainfall anomaly (mm/d)
B Low Flood Years: June rainfall anomaly (mm/d)
C June 2016 rainfall anomaly (mm/d)
27N
26.5N
26N
25.5N
25N
24.5N
24N
23.5N
23N
22.5N
22N
21.5N
21N
87E
87.5E
88E
88.5E
89E
89.5E
90E
90.5E
91E
91.5E
92E
92.5E
93E
-10
-9
-8
-7
-6
-5
-4
-3
-2
-1
1
2
3
4
5
6
7
8
9
10
