# Supplementary material for: Cholera forecast for Dhaka, Bangladesh, with the 2015-2016 El Niño: Lessons learned
Source: PLoS One. 2017 Mar 2;12(3):e0172355. doi: 10.1371/journal.pone.0172355 (PMC5333828; doi:10.1371/journal.pone.0172355)
Supplement: S1 Table — (DOCX) [file pone.0172355.s007.docx]

| **Parameter** | **Value at MLE** | **Confidence Intervals** |
| --- | --- | --- |
| Fixed infection - environmental reservoir ($\omega$) | 0.011 | 0 – 0.205 |
| Duration of infection (1 / $\text{γ}$) | 5 days | 3 – 12 days |
| Duration of immunity (1 / $\phi$) | 14 days | 0 – 36 days |
| Reporting rate ($\rho$) | 5.8 10^-5^ | 1.7 10^-5^ – 9.6 10^-5^ |
| b4E | 16 | 1.5 – 67 |
| b5E | 14 | 0.5 – 52.5 |
| Long term trend ($\nu$) | 0.005 | 0.0046 – 0.0145 |
