# Supplementary material for: Cholera forecast for Dhaka, Bangladesh, with the 2015-2016 El Niño: Lessons learned
Source: PLoS One. 2017 Mar 2;12(3):e0172355. doi: 10.1371/journal.pone.0172355 (PMC5333828; doi:10.1371/journal.pone.0172355)
Supplement: S1 Text — (DOCX) [file pone.0172355.s008.docx]

**Supporting Information**

**Cholera forecast for Dhaka, Bangladesh, with the 2015-2016 El Niño: lessons learned**

Pamela P. Martinez, Robert C. Reiner Jr., Benjamin A. Cash, Xavier Rodó, Mohammad Shahjahan Mondal, Manojit Roy, Mohammad Yunus, A.S.G. Faruque, Sayeeda Huq, Aaron A. King, and Mercedes Pascual.

**Mechanistic model**

The population dynamics of cholera are represented by an SIRS (Susceptible-Infected-Recovered-Susceptible) model (equation 1) in which the population in subdivided into the following classes: $S$ for naïve individuals who are susceptible to disease, $I$ for infected and infectious individuals, and $R$ for those who have recovered and have acquired immunity to the disease. This representation allows for temporary immunity with individuals in *R* eventually returning to S at a given rate $\phi$. The set of equations is given by:

$$\frac{dS}{dt} =\left( \mu P+ \frac{dP}{dt} \right)+\phi R-\lambda(t)S-\mu S,$$

$$\frac{dI}{dt} =\lambda\left( t \right)S-\gamma I-\mu I, (1)$$

$$\frac{dR}{dt}=\gamma I-\phi R-\mu R,$$

where *P* represents the population size, and *S*, *I* and *R*, the number of individuals in those respective classes. The force of infection $\lambda$ denotes the rate of infection per individual susceptible in the population, which is given by:

$$\lambda\left( t \right)=e^{-\nu\left( t-t_{0} \right)}\left[ \beta\left( t \right)\frac{I}{N}+\omega\right] (2)$$

where the first term implements a long-term trend starting at the initial time to, and in the second term, $\omega$ represents a fixed background infection due to an environmental reservoir and $\beta$ refers to the transmission rate per infection:

$$\beta\left( t \right)=exp\left[ \sum_{k=1}^{6} b_{j}s_{j}\left( t \right)+ \sum_{m=4}^{5} b_{Em}s_{m}\left( t \right)f\left( ENSO \right) \right]\left[ \frac{d\Gamma}{dt} \right] (3)$$

The expression for the transmission rate $\beta$ includes a seasonal component implemented through six splines $s_{j}$ with six coefficients $b_{j}$ determining the weight of each component, allowing for a flexible representation of the seasonality (S6A Fig). Climate forcing by ENSO is incorporated by considering the SST anomalies in the Nino3.4 region of the subtropical Pacific for the month of January (http://www.cpc.ncep.noaa.gov/data/indices/sstoi.indices). Specifically, this covariate is included in the terms relevant to the fall months (fourth and fifth splines), based on previous studies of the observed correlations between ENSO and cholera cases in this region [1,2]. The transmission rate also includes a stochastic component (through a Gamma distribution Γ) to represent environmental or other sources of variation not accounted for by seasonality or ENSO.

Following Reiner et al. (2012) [3], the El Niño index is integrated into a sigmoidal function, where ${ENSO}_{Jan}$ refers to the anomaly of the ENSO reported for January at time $t$, normalized between -1 and 1 (S6B Fig). This functional form allows for nonlinear responses to ENSO and is given by the following expression:

$$f\left( ENSO \right)=A\frac{\text{tan}\text{ }\left( h{ENSO}_{Jan}(t) \right)}{\text{tan}\text{ }\left( h \right)} (4)$$

We formally relate the model to data by assuming that only a fraction *ρ* of new infections are detected by the surveillance methods (*ρ* is estimated from data along with other model parameters, see below), and model the data as negative binomially distributed around these new infections, as follows. From equation 1, the rate of new infections is $\lambda S$, which gives the total new infections $\Delta I_{k}$ over a time interval $\left( t_{k-1},t_{k} \right)$ as $\Delta I_{k}=\rho\int_{t_{k-1}}^{t_{k}} \lambda S\left( t \right)dt$, and the measurement model that couples $\Delta I_{k}$ to the observed data $y_{k}$ at time $t_{k}$ is given as $y_{k}\sim\text{NegBin}\left( \Delta I_{k},\sigma_{\text{obs}}^{2} \right)$, where NegBin(*a*,*b*) is the negative binomial distribution with mean *a* and variance *a* + *a*^2^*b*, and $\sigma_{\text{obs}}^{2}$ denotes the variance parameter associated with detection uncertainty.

**Parameter estimation**

To evaluate the forecasting abilities of the model, we first fitted the model to the data between 1995-2010. Specifically, we carried out a likelihood-based inference via an iterated particle filtering method to estimate the parameters and initial conditions, to obtain the MLE (Maximum Likelihood Estimates) [4-6]. Because we wish to reproduce as close as possible the conditions under which one would be generating the forecasts, we then updated the estimated parameters by extending the fit of the model to the ‘new’ data that would have become available each year. This resulted in refining the fit of the model over a moving window of 12 months, starting from January 2011. The updated parameters and initial values of the state variables each January were used to simulate forward for the next year.

**Prediction evaluation**

To compare the predictions to the data for the years between 2011 and 2015, we first defined an outbreak as a season in which the total number of cases exceeds a given threshold. The threshold itself was determined based on the empirical distribution of the average number of cases between August and December for the period 1995-2010. Based on the predictions generated by 1000 simulations, we computed the predicted probability of surpassing the given threshold (top 50%, 25% and 5% of all of the data). These respective probabilities can be interpreted as an outbreak, large outbreak and extreme outbreak respectively.

**References**

1. Pascual M, Rodó X, Ellner SP, Colwell R, Bouma MJ. Cholera dynamics and El Niño-southern oscillation. Science. 2000; 289(5485): 1766–9.

2. Bouma MJ, Pascual M. Seasonal and interannual cycles of endemic cholera in Bengal 1891--1940 in relation to climate and geography. The Ecology and Etiology of Newly Emerging Marine Diseases. Springer; 2001. pp. 147–56.

3. Reiner RC, King AA, Emch M, Yunus M, Faruque ASG, Pascual M. Highly localized sensitivity to climate forcing drives endemic cholera in a megacity. Proc Natl Acad Sci. 2012; 109(6): 2033–6.

4. Ionides EL, Bretó C, King AA. Inference for nonlinear dynamical systems. Proc Natl Acad Sci. 2006; 103(49): 18438–43.

5. King AA, D N, Ionides EL. Statistical Inference for Partially Observed Markov Processes via the R Package pomp. J Stat Softw. 2016; 69(1): 1–43.

6. King AA, Ionides EL, Breto CM, Ellner SP, Ferrari MJ, Kendall BE, Lavine M, Nguyen D, Reuman DC, Wearing H, Wood SN. pomp: Statistical Inference for Partially Observed Markov Processes (R package, version 1.5). 2016. http://kingaa.github.io/pomp.
